# Supplementary material for: Continuous-variable tomography of solitary electrons
Source: Nat Commun. 2019 Nov 22;10:5298. doi: 10.1038/s41467-019-13222-1 (PMC6874662; doi:10.1038/s41467-019-13222-1)
Supplement: Supplementary file 1 — Supplementary Information [file 41467_2019_13222_MOESM1_ESM.pdf]

Supplementary Material: Continuous-Variable Tomography of Solitary Electrons

Fletcher *et al.*

Supplementary Note 1: Absolute energy scale and inelastic effects

Electrons arrive at the energy-selective barrier with an energy of order $E_0 \sim 100$ meV above the Fermi energy, set by the height of the pump exit barrier controlled by V_{G2}[1]. Approximately 25 meV is kinetic energy due to the cyclotron motion and drift motion and the rest is a potential energy (the electrons travel in a region of elevated potential at the edge). Determining the absolute energy requires a separate calibration procedure, for instance through voltage biasing the input to the electron pump[1,2]. In contrast, relative energy measurements can be easily calibrated using the fact that a detectable number of electrons emit optical phonons, and that this energy loss is well known[3]. Emission of longitudinal optical (LO) phonons[1,4] is almost entirely suppressed here using edge depletion gates (here $V_{G4} \simeq -0.25\ V$) under which the hot electrons travel[5] such that 95%+ electrons traverse the path from source to the energy-selective barrier without energy loss[4,5], but there is a small residual feature that enables a barrier calibration. Supplementary Figure. 1 shows the threshold V_{G3}^{DC} to block electrons at the energy selective barrier which depends linearly on V_{G2}. There is a replica of this feature corresponding to the electrons which have emitted a phonon[6] i.e. a known loss of $\Delta E_{LO} = \hbar\omega_{LO} \simeq 36$ meV, is $\Delta V_{G3} \simeq 59 \pm 2$ mV higher in gate voltage, from which the calibration factor $\alpha_h = \Delta E_{LO}/\Delta V_{G3} = (0.61 \pm 0.02)$ meV/mV can be found, similar to previous work[1,7].

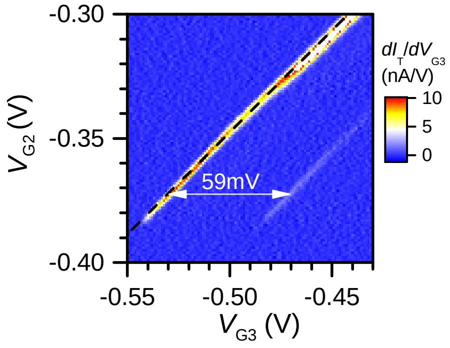

Supplementary Figure 1: Static barrier energy spectroscopy. Dashed line shows the linear dependence of the blocking threshold setting of V_{G3} with exit barrier voltage V_{G2}. The arrow shows how much lower the energy selective barrier voltage must be lowered before electrons that have emitted an LO phonon contribute to I_T. This enables a calibration of the relative barrier height.

Supplementary Note 2: Back-projection Algorithm

Data for a given back projection is gathered by first identifying the value of V_{G3} around which arrival energy is centred (using a static barrier) and then the approximate time of arrival (with a step-like barrier waveform). This gives value of $V_{\mathrm{G3}}^{\mathrm{DC}}$ and Δt_d through around which various scans are performed for different values of θ. We step both $V_{\mathrm{G3}}^{\mathrm{DC}}$ and Δt_d in increments of ~ 1.1 ps and 0.25 mV, over a range of 180 ps and 40 mV respectively through this centre coordinate, reversing the sign of the time sweep for $\theta < 0°$. The data measured along this measurement line S' is remapped onto the projection axis S. A two-dimensional filtered back-projection scheme is applied to the resulting sinogram. This can be performed with *Mathematica* function *InverseRadonTransform*[8] or can be evaluated manually as in Supplementary Figure 2 giving almost identical results. Each pixel in the output map has a coordinate x_i, y_j indexed by i, j. Each output values is evaluated from the integral of a sinusoidal trajectory in the sinogram, as shown in Supplementary Figure 2, which is indexed using the polar coordinates $|r|$ and ϕ, measured from the centre of the backprojection. As the sinogram is a discrete dataset, indexed by angular coordinate θ_m and detector axis coordinate s_n, interpolated values between near pixels (e.g. $n_{i,j}$ and $n_{i,j}+1$) are summed along the trajectory to give the backprojection output. It is well known that accurate reconstructions require a linear high-pass filter to be applied to the data before integration[9]. This can be performed in the frequency domain with an optional low pass behaviour at the very highest frequency (e.g. a Hann filter, as used here) to suppress noise. Numerical backprojection schemes can display artefacts (e.g. streaks) if the discrete gridding and angular resolution are not high enough (this is a weak effect here).

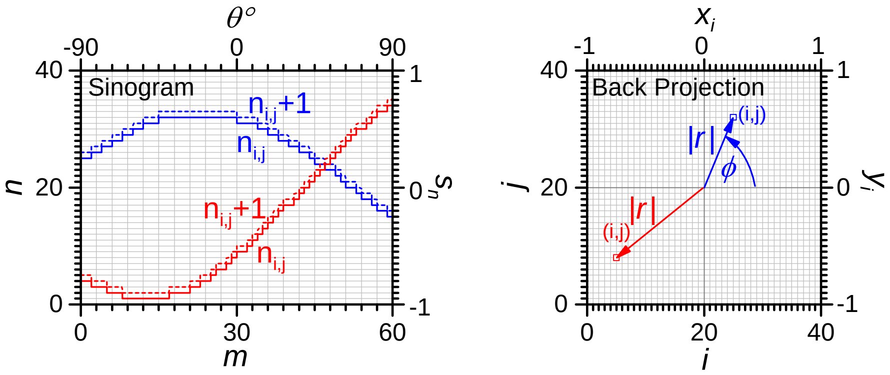

Supplementary Figure 2: Backprojection procedure. Illustration of discrete sinogram (left) coordinates and the trajectories along which a sum is evaluated to give back-projection (right, pixel granularity is exaggerated by a factor ~ 4 for illustration). Two example trajectories are shown (coloured red and blue) corresponding to the pixels in the backprojection. A sum is taken over the linear interpolation of the two nearest sinogram pixels (solid and dashed lines).

Supplementary Note 3: Waveform Synthesis and Transmission Maps

Examples of programmed energy barrier waveforms are illustrated in Supplementary Figure 3 for $\theta = -90°, -75°...+90°$. The relevant region for tomography is the region in the centre, indicated with a grey rectangle. We measure the resulting ramp rate $\beta_E(\theta)$ where $\beta_0 = \beta_E/\tan(\theta)$ using the technique from Ref.10. Examples of the transmitted current are shown in Supplementary Figure 4 for various angles. The trajectories corresponding to the projection axis S and the experimental measurement axes S' are illustrated with white arrows (see Supplementary Note 2). The slope of the linear ramp β_E (extracted from the constant current lines) changes as expected with an accurracy of better than 4% at rotation angles up to approximately $\theta \gtrsim 86°$ where the energy selective barrier channel rf bandwidth limits $dV_{G3}/dt \lesssim 3$ mV/ps. Beyond this the ramp-rate begins to saturate and falls short of the limit $dV_{G3}/dt \to \infty$ for $\theta = \pm 90°$. This sets a limit on resolving very sharp features e.g. extremely fast chirps (the chirp seen here is well within these limits).

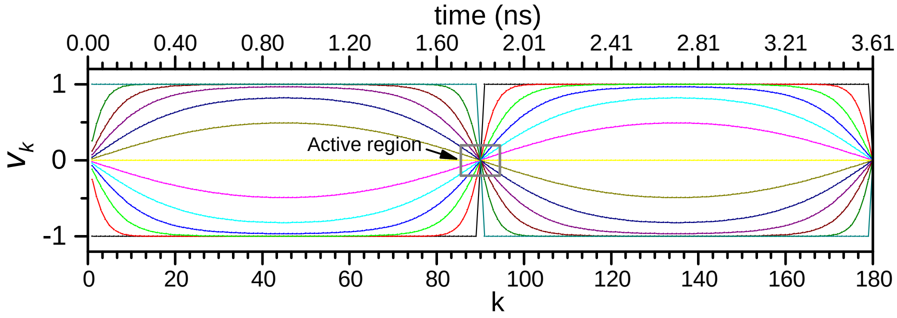

Supplementary Figure 3: Waveform Synthesis. Example waveforms calculated using the expression in the methods section for angles θ in 15° increments. The active part of the waveform is the zero crossing (indicated) which can be synchronised with electron arrival time.

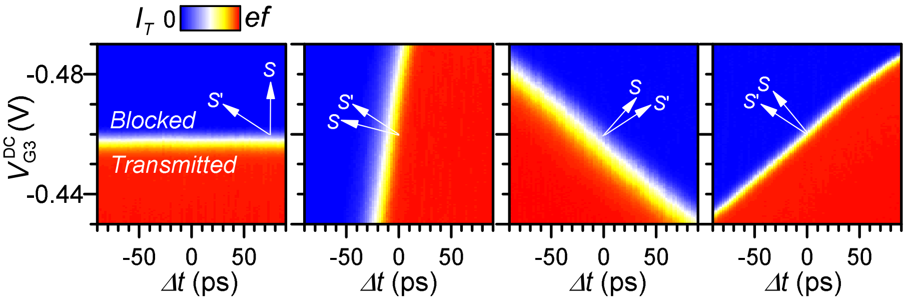

Supplementary Figure 4: Transmitted Current Maps. Measured transmitted current I_T at selected waveform projection angles θ for small offsets in the threshold barrier waveform time delay Δt and voltage offset V_{G3}^{DC}. At each angle the white arrow indicates the relevant trajectory for the sinogram.

Supplementary Note 4: Semi-classical model

We adapt a semiclassical model[7,11,12] for the ejection dynamics of a tunable-barrier quantum dot. The emission rate $\Gamma(t)$ and the energy level $\varepsilon(t)$ are related exponentially via,

$$\Gamma(t) = \Gamma_0 \exp\left[\varepsilon(t)/\Delta_{\rm ptb}\right], \tag{1}$$

where the energy sensitivity of the tunnel barrier is captured by an energy scale $\Delta_{\rm ptb}$, Γ_0 sets the overall tunneling rate and the dot energy ε is controlled by the external gate voltage $V_{\rm G1}$

$$\varepsilon(t) = -\alpha_p[V_{\rm G1}^{\rm DC} + V_{\rm G1}(t)] + \varepsilon_0, \tag{2}$$

via the lever arm α_p (the overall energy scale ε_0 controls the effective pump exit barrier height). The time-dependent occupation probability $P(t) = \exp[-\int^t \Gamma(t')\,dt']$ (initialised to $P = 1$ by the much earlier loading process) determines the distribution of emission times, $-dP(t)/dt$. The modulation of $\varepsilon(t)$ (dashed line), the increasing tunneling rate $\Gamma(t)$ (arrows) and the time dependence of the occupation probability $P(t)$ (shaded dots) are illustrated schematically in Supplementary Figure 5.

Without any energy broadening, the emission energy at time t is simply $\varepsilon(t)$, and the corresponding density in phase space is distributed along a sharp line (the emitter's classical trajectory[12]). Assuming energy smearing ΔE due to $T(E)$ to dominate over quantum broadening, we model the Wigner distribution as

$$W^{\rm model}(E,t) = -\frac{dT[\epsilon(t)]}{dE}\frac{dP(t)}{dt}, \tag{3}$$

with $dT(E)/dE = e^{-E^2/(2\Delta E^2)}/(\Delta E\sqrt{2\pi})$. The maximal width $\Delta E = 0.80$ meV is estimated by fitting $dT(E)/dE$ to the slice of experimentally measured density along the energy axis, $W(E,t_0)$, at the slowest emission conditions. For estimation of the rest of the model parameters, the sinusoidal time dependence of $V_{\rm G1}(t)$ is approximated around the extremum (with negligible loss of accuracy) as $V_{\rm G1}(t) = A(2\pi f t)^2/2$, resulting in a closed form analytic expression for $P(t)$. The parameters α_p, $\Delta_{\rm ptb}$, A, and $\Gamma_0\exp(\varepsilon_0/\Delta_{\rm ptb})$ are determined by a simultaneous fit to the experimentally determined mean emission time t_0 and distribution widths σ_t and σ_E as functions of $V_{\rm G1}^{\rm DC}$. The resulting model distributions $W^{\rm model}(E,t)$ are shown in Supplementary Figure 6, to be compared with the experimental reconstructions $W(E,t)$ in Fig. 3 of the main paper. Good agreement (with $\alpha_p = 0.50$ meV/mV and $\Delta_{\rm ptb} = 1.2$ meV) for slow emission rates justifies the neglect of quantum broadening effects in the model. The fitted model is also consistent with incomplete emission (a 8% reduction in pumped current) for the slowest emission.

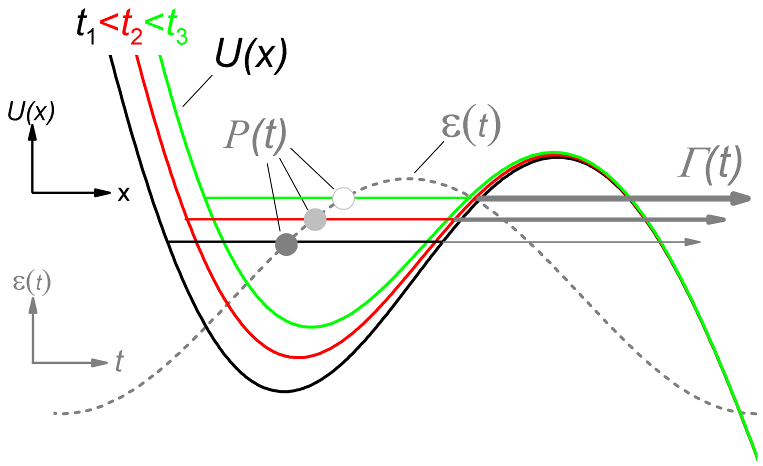

Supplementary Figure 5: Schematic representation of semiclassical emission dynamics. Semiclassical emission model from dynamical single quantum dot[7] with chemical potential $\varepsilon(t)$ with dot occupation probability $P(t)$ (schematically represented by shaded dots) by unloading into an empty states with a tunneling rate $\Gamma(t)$ (grey arrows) shown in three time steps $n = 1, 2, 3$.

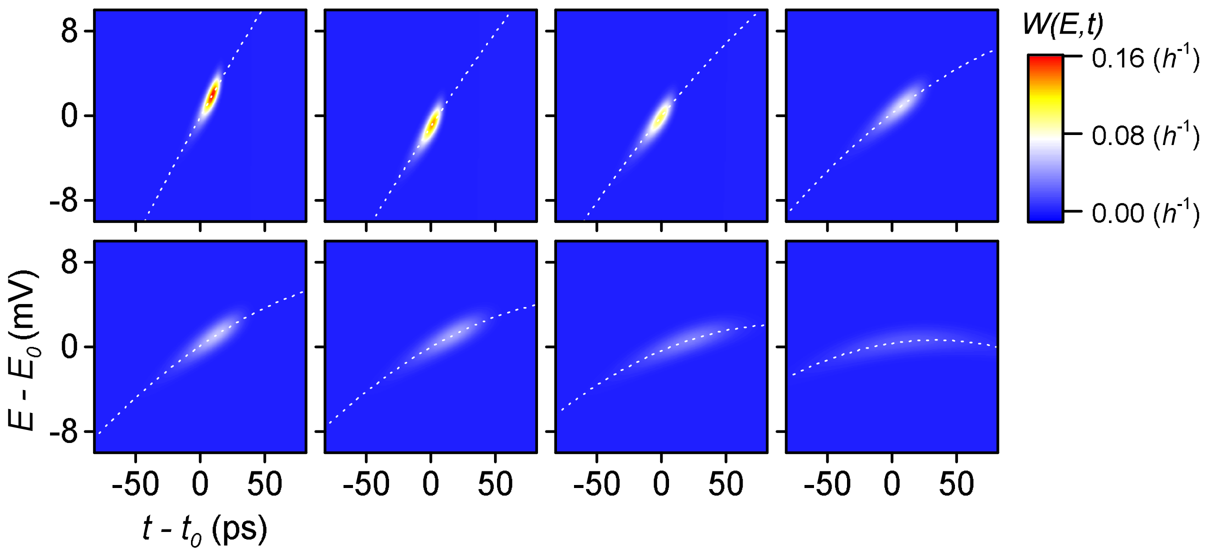

Supplementary Figure 6: Semiclassical model calculation. Solution $W^{\mathrm{model}}(E,t)$ to semiclassical ejection model in the presence of additional energy broadening. Model parameters are chosen to match those in Fig. 3c. Lines are the trajectory of $\varepsilon(t)$.

Supplementary Note 5: Analysis of distribution shape

For analysis of the distribution shape and size it is useful to parameterise the width of the distribution projected in either energy (σ_E) or time (σ_t) and to evaluate the energy time correlation. Supplementary Figure 7 shows projections and their widths for the eight different ejection speeds in time (upper panels) and energy (lower panels). Fits of the projected widths to the semiclassical model are shown.

To estimate the energy time correlation we fit the 2D data to bivariate normal distribution, given by

$$\rho(E,t) = \frac{1}{2\pi\sigma_E\sigma_t\sqrt{1-r^2}} \exp\left(-\frac{(E/\sigma_E)^2 - 2rEt/(\sigma_t\sigma_E) + (t/\sigma_t)^2}{2(1-r^2)}\right), \quad (4)$$

where E and t are measured with respect to a centre fit coordinate (t_0, E_0). This gives a correlation coefficient in the range $r \simeq 0.75 \pm 0.1$ in all but the quasi-static case where $(dV_{\mathrm{G1}}/dt \to 0)$. Information about the phase space density can be extracted from the characteristic widths of the distribution, whose product should not violate the Heisenberg uncertainty principle $\sigma_E\sigma_t/(\hbar/2) > 1$. Spread of the wave-packet due to non-static dot energy during emission has a major effect on this, which we can estimate.

Energy broadening ΔE present in the barrier function $T(E)$ gives larger apparent $\sigma_E = \sqrt{(\Delta E)^2 + \sigma_E'^2}$ than would be measured if $\Delta E \to 0$. By estimating $\Delta E = 0.8$ meV from ejection under near-static conditions we can then correct for the broadening to get $\sigma_E' = \sqrt{\sigma_E^2 - \Delta E^2}$. We cannot rule out similar effects of time broadening on σ_t, but these are more difficult to quantify. This gives $2\sigma_E'\sigma_t/\hbar \gtrsim 27$ at all ejection speeds. This is strongly influenced by the movement of the dot energy on scales much larger than the quantum broadening, as in the semiclassical model above. For a linearly correlated Gaussian distribution the Heisenberg uncertainty becomes $\sigma_t\sigma_E \geq \hbar/(2\sqrt{1-r^2})$ where r is the correlation coefficient[12]. Using the measured values of r we find $\chi = \sigma_t\sigma_E[\hbar/(2\sqrt{1-r^2})]^{-1} \gtrsim 27$ due to reduction by non-coherent broadening effects, such as finite ΔE. This is in agreement with the extracted values of γ, which for the Gaussian density (4) are related by $\gamma = 1/\chi$ (see Supplementary Note 6).

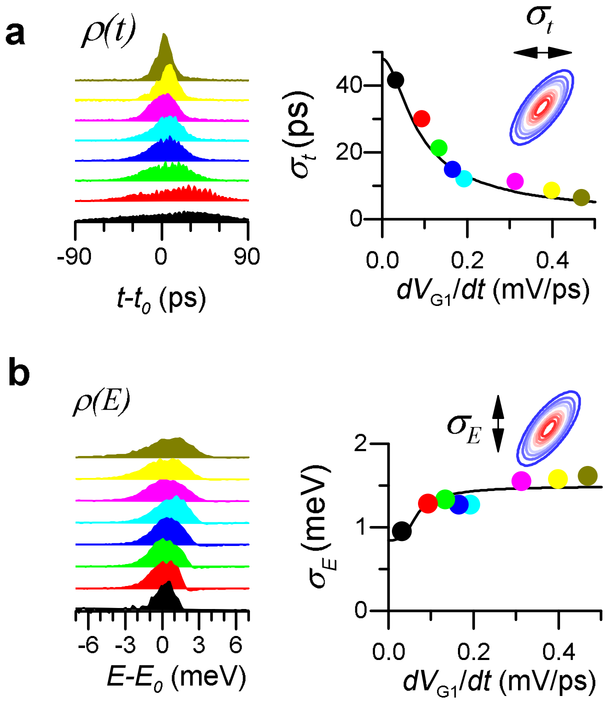

Supplementary Figure 7: Projected time and energy distributions and their widths. Upper panels (**a**), Projected time profile of the distributions (left, color fill matches dots in Fig. 3 of the main paper) and the measured temporal widths (right) at different pump barrier sweep rate. Line is fit to the numerical model described in the text. Lower panels (**b**), as above but showing energy projections.

Supplementary Note 6: Density matrix eigenvalues and state purity

To compute estimates of P_1 and γ we compute matrix representation of the density operator

$$\langle t|\hat{\rho}|t'\rangle = \int W\left(\frac{t+t'}{2}, E\right) e^{iE(t-t')/\hbar} dE \tag{5}$$

on the grid of sampled times by evaluating the integral in the *r.h.s.* with the backprojection data W as a discrete sum over sampled energies (step size $\delta E = 68\ \mu$eV), and cutting off Nyquist aliases by setting $\langle t|\hat{\rho}|t'\rangle = 0$ for $|t-t'| < h/(2\delta E) = 30$ ps. Numerical diagonalization of density matrices thus obtained for each backprojection produces the corresponding $\{P_i\}$ of pure state statistical weights. In Suplementary Table 1 we compare $P_1 = \max_i P_i \approx hW_{\max}$ and similarly show $\gamma = \sum_i P_i^2 = h \iint W^2 dE\, dt$ and the value of $1/\chi$ extracted from fits to the data. This shows good agreement in the different ways of evaluating P_1 and γ from the data.

dV/dt (mV/ps)	$\max_i P_i$	$hW_{\max}$	$\sum_i P_i^2$	$h\langle W\rangle$	$1/\chi$
0.43	0.070	0.074	0.039	0.037	0.036
0.36	0.067	0.069	0.036	0.035	0.033
0.29	0.056	0.057	0.030	0.029	0.027
0.18	0.055	0.058	0.028	0.027	0.027
0.16	0.046	0.049	0.024	0.024	0.023
0.14	0.034	0.035	0.018	0.018	0.016
0.09	0.027	0.028	0.013	0.013	0.011
0.02	0.020	0.019	0.010	0.009	0.008

Supplementary Table 1: Measures of state purity at different ejection speeds Maximum statistical weight of pure state $\max_i P_i$ is extracted by numerical diagonalisation to be compared with the peak density $hW_{\max}$ taken from backprojection. The purity of the effective mixed state $\sum_i P_i^2$ is also extracted by numerical diagonalisation, which is in agreement with the mean density $hW_{\max}$ from backprojection and $1/\chi$ evaluated from fits to the distribution.

Supplementary Note 7: Generality of technique over energy and time ranges

We consider the limitations imposed by expected typical experimental parameters: *Linear Sweep Range:* The energy and time range of the probed distribution is small enough to be encompassed by a linear region of the energy-selective barrier. For example, in our case the size of the voltage range scanned was typically 40 mV, well within the linear region of the signal applied to $V_{G3}(t)$. If the characteristic energy broadening of our source were larger, a larger AC amplitude may be required.

Proximity to Fermi Energy: The AC amplitude of $V_{G3}(t)$ should not be so large that the energy barrier drops below the Fermi energy - this would swamp the detector signal with the resulting large non-quantized current. Provided the appropriate signals are used, we expect the technique to work within any ranges near (but not including) the Fermi energy, up to the 100-200 meV range here[1].

Resolution and scaling: The scaling parameter β_0 sets the overall aspect ratio of the back projections, and is related to the temporal resolution. This number is partly chosen to reveal the characteristic features of the distribution being studied (given that there is always a discrete set of projection angles) and partly due to the bandwidth limit of the RF lines used to drive V_{G3}^{AC}. Although β_0 can be set arbitrarily to produce backprojections with a narrower time range, the back-projection becomes less accurate due to the absence of any data at large angles which correspond to physically inaccessible sweep rates. In our case the maximum β_E limits the effective upper projection angle to $|\theta| \lesssim 86°$ for our choice of β_0. Such angular range limitation effects have been seen in other systems[13] to complicate interpretation of the resulting backprojections. For the data here this does not have a gross effect on the shape of the distributions as the available data covers a wide enough range of angles, for instance encompassing the 'chirp' angle.

Supplementary References

1. Fletcher, J. D. *et al.* Clock-controlled emission of single-electron wave packets in a solid-state circuit. *Phys. Rev. Lett.* **111**, 216807 (2013).
2. Ubbelohde, N. *et al.* Partitioning of on-demand electron pairs. *Nature Nanotechnology* **10**, 46–49 (2014).
3. Sivan, U., Heiblum, M. & Umbach, C. P. Hot ballistic transport and phonon emission in a two-dimensional electron gas. *Phys. Rev. Lett.* **63**, 992–995 (1989).
4. Kataoka, M. *et al.* Time-of-flight measurements of single-electron wave packets in quantum hall edge states. *Phys. Rev. Lett.* **116**, 126803 (2016).
5. Johnson, N. *et al.* LO-Phonon emission rate of hot electrons from an on-demand single-electron source in a GaAs/AlGaAs heterostructure. *Phys. Rev. Lett.* **121**, 137703 (2018).
6. Taubert, D. *et al.* Relaxation of hot electrons in a degenerate two-dimensional electron system: Transition to one-dimensional scattering. *Phys. Rev. B* **83**, 235404 (2011).
7. Waldie, J. *et al.* Measurement and control of electron wave packets from a single-electron source. *Phys. Rev. B* **92**, 125305 (2015).
8. URL `https://reference.wolfram.com/language/ref/InverseRadonTransform.html`.
9. Smith, S. W. *The Scientists and Engineer's guide to Digital Signal Processing* (California Technical Publishing, 1997).
10. Johnson, N. *et al.* Ultrafast voltage sampling using single-electron wavepackets. *Applied Physics Letters* **110**, 102105 (2017).
11. Leicht, C. *et al.* Generation of energy selective excitations in quantum hall edge states. *Semiconductor Science and Technology* **26**, 055010 (2011).
12. Kashcheyevs, V. & Samuelsson, P. Classical-to-quantum crossover in electron on-demand emission. *Phys. Rev. B* **95**, 245424 (2017).
13. Kurtsiefer, C., Pfau, T. & Mlynek, J. Measurement of the Wigner function of an ensemble of helium atoms. *Nature* **386**, 150 (1997).
